# Effects of micronized bamboo powder on performance, nutrient utilization, intestinal histology, and cecal microbiota in laying hens

**DOI:** 10.3389/fmicb.2026.1865359

**Published:** 2026-06-18

**Authors:** Fawen Dai, Siqi Yang, Muqu Jin, Xia Huang, Qin Zhou, Tao Lin, Xiang Nong, Yaojun Yang, Jianjun Zuo

**Affiliations:** 1 College of Life Sciences, Leshan Normal University, Leshan, Sichuan, China; 2 Provincial Engineering and Technology Research Center for Innovative Development of Bamboo Fiber Nutrition, Leshan, Sichuan, China; 3 Bamboo Resource Conservation and Utilization Key Laboratory of Sichuan Province, Leshan, Sichuan, China; 4 College of Animal Science, South China Agricultural University, Guangzhou, Guangdong, China

**Keywords:** cecal microbiota, intestinal histology, laying hen, laying performance, micronized bamboo powder, nutrient digestibility

## Abstract

**Introduction:**

Bamboo clum powder is an unconventional feed ingredient rich in insoluble dietary fiber, and its potential application and optimal inclusion level in laying hen diets remain unclear. This study was conducted to evaluate the effects of dietary supplementation with micronized bamboo powder (MBP) on laying performance, apparent nutrient digestibility, intestinal morphology, and cecal microbiota in late-laying hens, and to determine its appropriate inclusion level.

**Methods:**

A total of 480 42-week-old Hy-Line Brown laying hens were randomly allocated into 4 groups with 5 replicates of 24 hens each. The trial hens were fed a basal diet (CON) or basal diets supplemented with 2% (TRE1), 3% (TRE2), or 4% (TRE3) MBP for 28 days following a 7-day adaptation period.

**Results:**

Compared with the CON group, the TRE3 group exhibited a significantly lower laying rate during days 15–28 and the overall experimental period of 1–28 d (*p* < 0.05), while no significant differences were observed between the TRE1, TRE2, and CON groups (*p* > 0.05). Moreover, the TRE3 group had significantly lower egg mass during days 15–28 and days 1–28 compared with the CON and TRE1 groups (*p* < 0.05). Treatments had no significant effect on serum triglyceride, total cholesterol, total protein or glucose levels (*p* > 0.05). The TRE3 group showed significantly lower apparent digestibility of dry matter, crude ash, crude protein, and gross energy compared to the CON group (*p* < 0.05). Intestinal histological analysis revealed that the TRE3 group had significantly greater duodenal and ileal wall thickness than the CON group (*p* < 0.05). Conversely, the TRE1 and TRE2 groups exhibited significantly higher jejunal villus height compared to the CON group (*p* < 0.05). 16S rRNA sequencing analysis of cecal microbiota indicated that dietary supplementation with MBP altered microbial diversity and composition. Specifically, the Sobs, Ace, and Chao indices were significantly lower in the TRE1 group than those in the CON group (*p* < 0.05). At the phylum level, the relative abundance of *Actinobacteriota* was significantly higher in the TRE1 and TRE3 groups that in the CON group (*p* < 0.05). At the genus level, the proportion of *WPS-2* in the TRE2 group was significantly higher than that in the CON group (*p* < 0.05), while the proportion of *Clostridia_UCG-014* in the TRE3 group was significantly lower than that in the CON group (*p* < 0.05). Correlation analysis further revealed that the abundances of *Ruminococcus_torques_group* and *NK4A214_group* (phylum *Firmicutes*) were significantly negatively correlated with daily feed intake, whereas *Blautia* showed a significant positive correlation with daily feed intake (*p* < 0.05). Moreover, the abundance of *NK4A214_group* was significantly negatively correlated with feed efficiency, while the abundance of *Olsenella* (phylum *Actinobacteriota*) was significantly positively correlated with feed efficiency (*p* < 0.05).

**Discussion:**

In conclusion, excessive supplementation of MBP (4%) led to thickened intestinal walls, decreased nutrient utilization, and reduced laying rate and egg mass in hens, which may be associated with a reduction in the abundance of beneficial cecal bacteria. Moderate MBP supplementation (2–3%) as a partial substitute for corn had no adverse effects on performance, despite some changes in digestibility and microbiota. This suggests that MBP has potential as an alternative feed ingredient for corn in laying hen diets, but its inclusion level requires careful consideration.

## Introduction

With the comprehensive ban on feed antibiotics, the beneficial role of fiber nutrition in modulating the poultry intestine has attracted widespread attention. The role of dietary fiber has been re-evaluated from its traditional perception as a “diluent” or “anti-nutritional factor” to a functional nutrient crucial for maintaining gastrointestinal health, regulating microbial homeostasis, and improving growth performance (Singh and Kim, 2021). Dietary fiber is composed of non-starch polysaccharides, oligosaccharides, and lignin, which can not be broken down or enzymatically digested during digestion. Based on its solubility in water, dietary fiber is classified into soluble dietary fiber (SDF) and insoluble dietary fiber (IDF). IDF supplemented in broiler diets can regulate intestinal morphology, digestive organ development, nutrient absorption, growth performance, and gut microbiota, while SDF is considered to increase intestinal digesta viscosity and is associated with negative changes in the gut microbiota and reduced nutrient absorption (Tejeda and Kim, 2021). However, due to factors such as diet formulation, fiber inclusion level, and fiber source, studies on lignocellulosic materials as IDF have reported highly variable effects on laying performance, nutrient digestibility, digestive tract development, and gut microbiota, with some studies even exhibiting negative effects (Röhe and Zentek, 2021). These findings indicate that in the post-antibiotic era, the application of fiber nutrition to improve the intestinal health of laying hens requires careful consideration of fiber characteristics and inclusion level, and its effects are associated with the regulation of the gut microbiota. Research has confirmed that the “microbiota-accessible fiber → gut microbiota → short-chain fatty acids/bile acid axis” plays a key role in regulating intestinal mucosal function in poultry (Yang et al., 2024). Therefore, the development of novel functional fiber ingredients should focus on investigating their regulatory effects on intestinal health and microbiota.

In practical production, traditional by-product fiber ingredients such as wheat bran and sugar beet pulp often face the risk of mycotoxin contamination, which can lead to liver lesions, reduced antioxidant capacity, and ultimately decreased laying performance in hens (Ma et al., 2025). Therefore, the developing of safe and novel fiber ingredients holds significant practical importance. In recent years, researchers have attempted to develop novel lignocellulosic fiber ingredients for use in laying hen production, which can improve fiber digestibility and short-chain fatty acids (SCFAs) production by increasing the relative abundance of fiber-degrading bacteria and *Lactobacillus* (Sun et al., 2022). Furthermore, the processing method of fiber ingredients also deserves attention. It has been shown that micronization of IDF ingredients such as sunflower hulls, sugarcane bagasse, and wheat bran significantly reduced ileal digesta viscosity and improved the apparent ileal digestibility of fat and organic matter in broilers (Pourazadi et al., 2020). Collectively, these studies provide technical references for the development of fiber ingredients for laying hens.

Bamboo is the natural food of giant pandas, and China, as the homeland of giant pandas, is rich in bamboo resources. Bamboo culms are rich in IDF, with total dietary fiber content ranging from 64.12 to 90.21%, representing a promising novel fiber ingredient (Felisberto et al., 2017). Currently, research on the application of bamboo fiber in monogastric livestock and poultry has made preliminary progress. Supplementation with 4% fermented bamboo fiber in the diet of sows during late gestation and lactation modulated the microbiota structure of both sows and piglets, enriching beneficial bacteria and reducing the abundance of harmful bacteria, thereby enhancing immune function, alleviating constipation and reducing backfat loss during lactation in sows, and improving piglet growth performance (Sun et al., 2023). Studies in broilers have also found that supplementation with appropriate levels of fermented bamboo fibers promoted intestinal tissue development and improved growth performance (Malyar et al., 2024). Our previous research found that supplementation with 1% micronized bamboo powder (MBP) in broiler diets significantly improved antioxidant capacity and feed efficiency, with the underlying mechanism primarily associated with the regulation of intestinal fatty acid, amino acid, and immune metabolic pathways (Dai et al., 2022). Collectively, these studies suggest that bamboo processed appropriately through methods such as micronization or fermentation, can be utilized in monogastric farm animals and may possess functional regulatory potential beyond mere physical “filling” effects.

However, systematic research on the application effects, optimal inclusion level, and underlying mechanisms of MBP in laying hens, particularly its dose–response effects on intestinal health and production performance in late-laying hens, is still lacking. Therefore, based on previous research, this study is conducted to investigate the effects of different levels (0, 2, 3, 4%) of MBP on the performance, serum biochemical parameters, nutrient apparent metabolizability, intestinal morphology, and cecal microbial diversity and composition in Hy-Line Brown laying hens.

We hypothesized that: moderate inclusion of MBP would improve intestinal morphology and modulate cecal microbiota without compromising laying performance or nutrient digestibility; excessive inclusion of MBP would induce intestinal wall thickening, reduce nutrient digestibility, and alter cecal microbial composition, ultimately leading to decreased laying performance; and the effects of MBP would be dose-dependent, with a safe upper limit of 3% inclusion in laying hen diets. The objective is to determine the appropriate inclusion level of MBP in laying hen diets and preliminarily explore its possible mechanism of action, thereby providing a theoretical basis for the scientific application of MBP as a novel, functional fiber resource in laying hen production.

## Materials and methods

### Ethics approval

The animal study was reviewed by Animal Care and Use Committee of Leshan Normal University (Certification No. 4151010649), China, and conducted in accordance with the approved protocol (No. LAC2023001). Written informed consent was obtained from the owners for the participation of their animals in this study.

### Preparation of MBP

*Phyllostachys edulis* clums, aged 5–6 years and harvested from Sichuan Province, China, were collected. After removing the outer layer (Huang et al., 2019), the clums were coarsely ground using a knife mill (Model 600, Zhengzhou Chuangyi Machinery Equipment Co., Ltd., China). The resulting coarse powder was dried to a moisture content of 12.50% and further ground using an impact mill (ZJ-C100, Sichuan Zhongjin Powder Equipment Co., Ltd., China) until it completely passed through a 200-mesh sieve. The particle size distribution of MBP was determined using a laser particle size analyzer, with a D_75_ was 22.42 μm. The crude fiber content of MBP (after the preparation, as a fed basis) was determined to be 39.96%, Ash 1.60%, NDF 70.41%, and total dietary fiber (TDF) 94.49%, IDF 94.01%, SDF 0.48%, according to Chinese national standards GB/T 6434–2022, GB/T 6438–2025, GB/T 20806–2022, and GB 5009.88–2023, respectively.

### Animals and experimental design

A total of 480 healthy 42-week-old Hy-Line Brown laying hens with similar body weight and production performance, were randomly allocated into 4 treatment groups. Each group consisted of 5 replicates, with 24 hens per replicate (each replicate comprised 6 adjacent cages, with 4 hens per cage). The control group (CON) was fed a basal diet, while treatment groups 1 (TRE1), 2 (TRE2), and 3 (TRE3) were fed the basal diet supplemented with 2, 3, and 4% MBP, respectively.

Hens were housed in a semi-open conventional hen house with a combination of artificial and natural light, ensuring 16 h of light and 8 h of darkness per day. During the experiment period, hens had ad libitum access to feed and water. The experiment lasted for 35 days, consisting of a 7-day adaptation period and a 28-day formal experimental period. During the adaptation period, all hens were fed a mixture of all four experimental diets in equal proportions (25% each), and average laying rate and average egg weight were recorded daily. The overall average laying rate of all groups during the adaptation period was 89.40 ± 1.96% (*p* = 0.636), and the average egg weight was 51.96 ± 0.22 g (*p* = 0.673).

### Experimental diets and nutrient levels

The experimental diets were formulated with reference to the *NRC Nutrient Requirements of Poultry* (NRC 1994) and the Chinese *Technical Specification for Layer Feed Formulation* (NY/T 33–2004). The ingredient composition and nutrient levels are shown in Table 1. All diets were prepared as mash.

**Table 1:** Composition and nutrient levels of the experimental diets (as-fed basis).

Items	CON	TRE1	TRE2	TRE3
	0%MBP	2%MBP	3%MBP	4%MBP
Ingredients (%)				
Corn	65.0	63.0	62.0	61.0
Soybean meal	25.0	25.0	25.0	25.0
Limestone	8.0	8.0	8.0	8.0
Soybean oil	1.0	1.0	1.0	1.0
Micronized bamboo powder (MBP)	-	2.0	3.0	4.0
Premix^1^	3.0	3.0	3.0	3.0
Total	100.0	100.0	100.0	100.0
Calculated nutrient levels				
ME (MJ/kg)	11.21	11.21	11.21	11.21
CP (%)	16.35	16.22	16.16	16.09
CF (%)	3.00	3.82	4.22	4.63
NDF (%)	11.05	12.50	13.22	13.94
Ca (%)	2.91	2.91	2.91	2.91
AP (%)	0.24	0.23	0.23	0.23
Lys (%)	0.73	0.72	0.72	0.72
Met + Cys (%)	0.54	0.53	0.53	0.53
Analyzed nutrient levels				
AME (MJ/kg)	10.95	10.60	9.56	9.46
CP (%)	16.46	16.31	16.25	16.44
EE (%)	2.94	2.90	2.91	2.96
Ash (%)	14.87	14.81	14.78	14.75

^1^The premix provided per kg of diet: Vit A 10,000 IU; Vit D_3_ 2,000 IU; Vit E 20 mg; Vit K_3_ 3 mg; Vit B_1_ 2.4 mg; Vit B_2_ 7.5 mg; Niacin 45 mg; D-pantothenic acid 12 mg; Vit B_6_ 3.6 mg; D-biotin 0.09 mg; Folic acid 1.2 mg; Vit B_12_ 15 μg; Fe 100 mg; Cu 8 mg; Zn 90 mg; Mn 90 mg; I 0.8 mg; Se 0.3 mg; 98.5% Methionine 100 mg; 50% Choline 2.6 g; Phytase 1,000 IU.

### Sample collection

A metabolism trial was conducted using the exogenous indicator method starting 8 days before the end of the experiment. Chromium oxide (Cr_2_O_3_) was added to the experimental diets at a level of 0.25% as an inert indicator. Fecal collection began on the 5th day of the metabolic trial, and fresh fecal samples were collected daily from each replicate for three consecutive days. Collection trays were placed under the cages to collect feces, which were carefully cleaned of feathers, dander, and other debris before being stored at −20 °C. After the collection period, fecal samples from each replicate were pooled and mixed thoroughly, dried at 65 °C for 72 h, ground to pass through a 40-mesh sieve, and stored at 4 °C for subsequent analysis.

At the end of the experiment, two hens from each replicate with body weights close to the replicate average were selected. Blood samples were collected from the wing vein into non-anticoagulant tubes, allowed to stand at room temperature for 30 min, and then centrifuged at 3,000 × g for 10 min at 4 °C. The serum was separated and stored at −20 °C for subsequent analysis of serum biochemical parameters. After blood collection, the selected hens were euthanized by exsanguination. Tissue samples of approximately 0.5 cm in length were collected from the middle sections of the duodenum, jejunum, and ileum. The samples were gently rinsed with pre-chilled phosphate-buffered saline (PBS) to remove digesta, blotted dry with filter paper, and immediately fixed in 4% paraformaldehyde solution for histological analysis. Following dissection, approximately 200 mg of cecal digesta was aseptically collected, placed into sterile 1.5 mL cryotubes, snap-frozen in liquid nitrogen, and stored at −80 °C for microbiome analysis.

### Laying performance

On a replicate basis, feed intake, egg number, and egg weight were recorded daily. Average daily feed intake (ADFI), laying rate, egg mass and feed efficiency were calculated for the periods of 1–14 d, 15–28 d, and 1–28 d of the experiment.

### Serum biochemical indices

Commercial kits manufactured by Nanjing Jiancheng Bioengineering Institute (Nanjing, China) were used to determine serum total protein (A045-4), glucose (A154-1-1), triglyceride (A110-1-1), and total cholesterol (A111-1-1). All procedures were performed strictly in accordance with the kit instructions. Total protein content was determined using the BCA microplate method, glucose using the hexokinase method, and triglycerides and total cholesterol using the single-reagent GPO-PAP enzymatic microplate method.

### Apparent nutrient digestibility

The chromium content in experimental diets and feces was determined determined using a wet digestion method followed by flame atomic absorption spectrophotometry (AA240, Agilent Technologies, USA) according to the GB/T 13088–2006 method. Briefly, samples were ashed at 600 °C for 5 h, digested with nitric acid solution, and the absorbance was measured at 370 nm.

Dry matter (DM) was analyzed according to GB/T 6435–2014, ash according to GB/T 6438–2007, crude protein (CP) according to GB/T 6432–2018, and crude fat (EE) according to GB/T 6433–2006. Gross energy (GE) was measured using an automatic oxygen bomb calorimeter (IKA C2000, Germany). The apparent nutrient digestibility was calculated using the following formula:

$\begin{array}{l} \text{Apparent digestibility}(\%)=100\%\\ -(\begin{array}{l} \text{nutrient content in feces}/\\ \text{nutrient content in diet} \end{array})\\ \times (\begin{array}{l} \text{indicator content in diet}/\\ \text{indicator content in feces} \end{array})\times 100\%. \end{array}$

The apparent metabolizable energy (AME) of each experimental diet was calculated using the following formula:

$\mathrm{AME}\;(\mathrm{MJ}/\mathrm{kg})=\mathrm{GE}\;\text{in diet}–(\begin{array}{l} \mathrm{GE}\;\text{in feces}\\ \times \text{indicator content in diet}/\\ \text{indicator content in feces} \end{array}).$

### Intestinal histomorphology analysis

Fixed intestinal tissue samples were dehydrated, embedded in paraffin, and sectioned into 5 μm thick slices, which were then stained with hematoxylin and eosin (HE). Images of the stained sections were captured using an optical microscope equipped with a digital imaging system (BA210 Digital, Motic, China). Measurements were performed on each section using image analysis software (Image-Pro Plus version 6.0). For each intestinal segment, villus height (from the villus tip to the crypt opening), villus width, crypt depth (from the crypt base to the crypt opening), and intestinal wall thickness were measured. Ten intact and well-oriented villi with their associated crypts were selected for measurement per sample, and the average values were calculated. The ratio of villus height to crypt depth (VH/CD) was subsequently calculated.

### Cecal digesta microbiota analysis

Genomic DNA was extracted from cecal digesta samples using a DNA extraction kit (Omega Bio-tek, Norcross, GA, USA) following the manufacturer’s instructions. The quality of extracted genomic DNA was assessed by 1% agarose gel electrophoresis, and DNA concentration and purity were determined using a NanoDrop 2000 spectrophotometer (Thermo Fisher Scientific, Inc., USA). Using the extracted DNA as a template, PCR amplification of the V3-V4 hypervariable region of the 16S rRNA gene was performed with primers 338F (5’-ACTCCTACGGGAGGCAGCAG-3′) and 806R (5’-GGACTACHVGGGTWTCTAAT-3′) (Liu et al., 2016). The resulting PCR products were subjected to paired-end sequencing on the Illumina MiSeq PE300 platform (Shanghai Majorbio Bio-Pharm Technology Co., Ltd., China). Downstream bioinformatic analyses were performed using the online platform of Majorbio Cloud Platform^1^ as previously described (Dai et al., 2024). The raw reads have been deposited into NCBI with project NO. PRJNA1430196.

Quality control of the raw paired-end sequencing reads was performed using fastp software^2^ (Chen et al., 2018) and reads were assembled using FLASH software^3^ (Magoč and Salzberg, 2011). Operational taxonomic units (OTUs) were clustered at 97% identity using UPARSE,^4^ and chimeras were removed. Sequences were rarefied to 34,060 per sample to standardize sequencing depth. Taxonomic annotation was performed using the RDP classifier against the Silva 16S rRNA database (Release 138), with a confidence threshold of 70%.

### Statistical analysis

Statistical analysis for laying performance, serum biochemical indices, and intestinal histological parameters was performed with the SPSS software package (version 23.0, IBM Corp., Armonk, NY, USA). The normality of the data was initially tested using the Shapiro–Wilk test. The data were then analyzed using one-way analysis of variance (ANOVA) and Orthogonal Polynomial Contrasts to determine linear and quadratic responses to different levels of MBP. Multiple comparisons were first conducted using Fisher’s least significant difference (LSD) test, and Tukey’s honestly significant difference (HSD) test was subsequently applied to verify the significance patterns. Results are presented as mean ± standard error (SEM). *p* < 0.05 was considered statistically significant, and *p* < 0.10 was considered a trend toward significance.

All 16S rRNA sequencing data analyses were conducted on the Majorbio Cloud Platform.^5^ Specifically, alpha diversity indices, including Ace, Chao, and Sobs for richness, and Shannon and Simpson indices for diversity, were calculated using mothur software.^6^ Differences in alpha diversity between groups were analyzed using the Wilcoxon rank-sum test. Principal coordinate analysis (PCoA) based on the Bray-Curtis distance algorithm was performed to examine the similarity of microbial community structure among samples, and the analysis of similarities (ANOSIM) was performed to test for significant differences in microbial community structure between groups. Differences in the relative abundance of major microorganisms at the phylum and genus levels between different experimental groups were analyzed using Student’s *t*-test (two-tailed), and the Benjamini-Hochberg false discovery rate (FDR) correction was automatically applied for multiple comparisons. Meanwhile, linear discriminant analysis effect size (LEfSe) was also performed to identify differentially abundant taxa with robust effect sizes. Spearman correlation analysis was applied to evaluate the correlations between top 20 genera and laying performance, intestinal morphology, and nutrient digestibility. For all microbiota data analyses, *p* < 0.05 was considered statistically significant.

## Results

### Effect of MBP on laying performance of hens

As shown in Table 2, laying rate and egg mass decreased (linear, *p* < 0.05) with increasing MBP supplementation during the later stage of the experiment (days 15–28) and the entire experimental period (days 1–28), whereas feed efficiency increased (linear, *p* < 0.05) in laying hens. Dietary MBP significantly influenced laying rate and egg mass (*p* < 0.05) during the later stage of the experiment (days 15–28). The laying rate in TRE3 group was significantly lower than that in CON, TRE1 and TRE2 groups (*p* < 0.05), and egg mass in the TRE3 group was significantly lower than that in CON and TRE1 groups (*p* < 0.05). For the entire experimental period (days 1–28), the TRE3 group exhibited significantly lower laying rate and egg mass compared with the CON, TRE1 and TRE2 groups (*p* < 0.05), whereas no significant differences were observed between the TRE1 or TRE2 groups and the CON group (*p* > 0.05). No significant differences were detected among groups in average daily feed intake or feed efficiency at any stage of the experiment (*p* > 0.05).

**Table 2:** Effect of MBP on laying performance of hens.

Items	CON	TRE1	TRE2	TRE3	SEM	*p*-value		
	0%MBP	2%MBP	3%MBP	4%MBP		ANOVA	Linear	Quadratic
Days 1–14								
ADFI (g/d)	104.17 ± 2.20	105.26 ± 2.17	104.97 ± 1.24	102.81 ± 3.47	2.41	0.401	0.378	0.149
Laying rate (%)	93.22 ± 2.65	94.28 ± 4.96	95.00 ± 2.65	86.43 ± 10.07	6.46	0.126	0.116	0.087
Egg mass (g/hen/d)	49.96 ± 2.02	50.08 ± 2.62	50.95 ± 1.28	47.34 ± 5.20	3.21	0.332	0.284	0.204
Feed efficiency (g/g)	2.09 ± 0.06	2.11 ± 0.10	2.06 ± 0.06	2.20 ± 0.27	0.15	0.534	0.360	0.398
Days 15–28								
ADFI (g/d)	94.26 ± 4.78	93.51 ± 6.07	95.81 ± 4.54	90.94 ± 10.79	6.68	0.741	0.593	0.521
Laying rate (%)	93.21 ± 3.43^a^	93.93 ± 3.24^a^	92.14 ± 5.14^a^	81.07 ± 9.99^b^	7.76	0.013	0.006	0.046
Egg mass (g/hen/d)	50.76 ± 3.09^a^	49.74 ± 1.79^a^	48.90 ± 2.87^ab^	44.16 ± 5.40^b^	4.16	0.043	0.010	0.257
Feed efficiency (g/g)	1.86 ± 0.07	1.88 ± 0.07	1.96 ± 0.12	2.07 ± 0.29	0.17	0.189	0.040	0.549
Days 1–28								
ADFI (g/d)	99.21 ± 3.42	99.38 ± 3.91	100.39 ± 2.27	96.87 ± 4.81	3.66	0.511	0.431	0.283
Laying rate (%)	93.21 ± 2.79^a^	94.11 ± 3.82^a^	93.57 ± 2.48^a^	83.75 ± 9.35^b^	6.62	0.022	0.016	0.041
Egg mass (g/hen/d)	50.36 ± 2.52^a^	49.91 ± 2.18^a^	49.93 ± 1.40^a^	45.75 ± 4.90^b^	3.39	0.093	0.037	0.190
Feed efficiency (g/g)	1.97 ± 0.06	1.99 ± 0.04	2.01 ± 0.08	2.13 ± 0.19	0.12	0.145	0.039	0.340

^a,b^Means in the same row with different superscripts differ significantly (*p* < 0.05). Values are mean ± SE, *n* = 5 replicates per treatment.

### Effect of MBP on serum biochemical indices of hens

As presented in Table 3, dietary supplementation with MBP had no significant effect on serum triglyceride, total cholesterol, total protein or glucose levels (*p* > 0.05). Serum glucose concentrations decreased (linear, *p* = 0.066) with increasing MBP supplementation. Compared with the CON group, the serum glucose concentrations in the TRE2 and TRE3 groups were numerically reduced by 10.75 and 21.05% (*p* > 0.05), respectively.

**Table 3:** Effect of MBP on serum biochemical indices of hens.

Items	CON	TRE1	TRE2	TRE3	SEM	*P*-value		
	0%MBP	2%MBP	3%MBP	4%MBP		ANOVA	Linear	Quadratic
TG (mmol/L)	7.83 ± 0.85	8.55 ± 1.37	8.23 ± 1.18	8.10 ± 1.25	0.57	0.854	0.964	0.867
TC (mmol/L)	1.97 ± 0.13	2.10 ± 0.18	2.50 ± 0.39	2.53 ± 0.27	0.13	0.326	0.089	0.841
TP (g/L)	52.62 ± 2.43	53.58 ± 1.44	56.44 ± 1.49	54.96 ± 1.59	0.88	0.472	0.590	0.442
GLU (mmol/L)	10.88 ± 0.76	10.51 ± 0.85	9.71 ± 0.83	8.59 ± 1.02	0.45	0.284	0.066	0.675

^a,b^Means in the same row with different superscripts differ significantly (*p* < 0.05). Values are mean ± SE, *n* = 10 hens per treatment.

### Effect of MBP on apparent nutrient digestibility of hens

As shown in Table 4, the effect of MBP on apparent nutrient digestibility was dose-dependent, apparent digestibility of DM (linear, *p* < 0.05), Ash (linear, *p* < 0.05), CP (linear, *p* = 0.068) and GE (linear, *p* < 0.05) decreased with increasing MBP supplementation. Dietary supplementation with MBP significantly affected the apparent digestibility of GE in hens (*p* < 0.05), which decreased linearly with increasing MBP inclusion. Compared with the CON group, the apparent digestibility of GE in the TRE2 and TRE3 groups decreased by 11.62 and 14.91%, respectively (*p* < 0.05), whereas no significant difference was observed between the TRE1 and CON groups (*p* > 0.05). Compared with the CON group, the TRE3 group also showed significant reductions in the apparent digestibility of DM, Ash, and CP, which decreased by 10.06% (*p* < 0.05), 18.85% (*p* < 0.05), and 21.35% (*p* < 0.05), respectively. In contrast, no significant differences in these parameters were observed between the TRE1 or TRE2 groups and the CON group (*p* > 0.05). There were no significant differences in the apparent digestibility of EE among any of the groups (*p* > 0.05).

**Table 4:** Effect of MBP on apparent nutrient digestibility of hens (%).

Items	CON	TRE1	TRE2	TRE3	SEM	*P*-value		
	0%MBP	2%MBP	3%MBP	4%MBP		ANOVA	Linear	Quadratic
DM	78.60 ± 1.85^a^	77.27 ± 1.29^a^	75.90 ± 0.61^ab^	70.69 ± 3.33^b^	1.16	0.067	0.020	0.198
Ash	57.77 ± 3.41^a^	56.13 ± 1.39^ab^	51.25 ± 1.67^ab^	46.88 ± 5.67^b^	1.87	0.153	0.036	0.403
CP	70.06 ± 4.89^a^	67.72 ± 3.21^ab^	69.91 ± 2.37^a^	55.10 ± 6.25^b^	2.48	0.089	0.068	0.141
EE	81.81 ± 2.76	79.29 ± 2.63	79.08 ± 2.79	79.70 ± 2.20	1.22	0.872	0.516	0.620
GE	74.44 ± 2.39^a^	72.72 ± 1.36^ab^	65.79 ± 1.91^bc^	63.34 ± 3.85^c^	1.58	0.019	0.004	0.369

^a−c^Means in the same row with different superscripts differ significantly (*p* < 0.05). Values are mean ± SE, *n* = 5 replicates per treatment.

### Effect of MBP on intestinal morphology of hens

As shown in Table 5, compared with the CON group, the TRE3 group exhibited a significant increase in duodenal intestinal wall thickness (*p* < 0.05). No significant differences were observed among groups in duodenal villus height, villus width, crypt depth, or the ratio of villus height to crypt depth (VH/VD) (*p* > 0.05).

**Table 5:** Effect of MBP on duodenal morphology of hens.

Items	CON	TRE1	TRE2	TRE3	SEM	*p*-value		
	0%MBP	2%MBP	3%MBP	4%MBP		ANOVA	Linear	Quadratic
Villus height (μm)	1175.33 ± 75.13	1237.31 ± 69.90	1124.21 ± 69.82	1130.95 ± 61.94	34.09	0.642	0.433	0.693
Villus width (μm)	184.95 ± 15.03	174.83 ± 7.59	169.90 ± 11.6	163.53 ± 6.35	5.30	0.552	0.158	0.862
Crypt depth (μm)	270.95 ± 12.30	301.65 ± 16.42	275.39 ± 12.35	298.85 ± 12.91	6.89	0.275	0.351	0.792
Intestinal wall thickness (μm)	319.12 ± 13.85^b^	360.67 ± 16.42^ab^	318.62 ± 21.18^b^	368.84 ± 14.28^a^	8.83	0.070	0.160	0.796
VH/CD	4.40 ± 0.32	4.22 ± 0.36	4.17 ± 0.33	3.84 ± 0.25	0.16	0.651	0.225	0.819

^a,b^Means in the same row with different superscripts differ significantly (*p* < 0.05). Values are mean, *n* = 10 hens per treatment.

As shown in Table 6, compared with the CON group, the TRE1 and TRE2 groups exhibited significantly increased jejunal villus height (*p* < 0.05), and the TRE2 group also showed significantly increased villus width (*p* < 0.05). Jejunal VH/VD ratio (linear, *p* < 0.05) increased with increasing MBP supplementation. The VH/VD ratio in the TRE2 and TRE3 groups was significantly higher than that in the CON group (*p* < 0.05). No significant differences were observed among groups in jejunal intestinal wall thickness (*p* > 0.05).

**Table 6:** Effect of MBP on jejunal morphology of hens.

Items	CON	TRE1	TRE2	TRE3	SEM	*P*-value		
	0%MBP	2%MBP	3%MBP	4%MBP		ANOVA	Linear	Quadratic
Villus height (μm)	991.29 ± 53.36^b^	1229.52 ± 44.86^a^	1170.96 ± 68.10^a^	1148.58 ± 73.65^ab^	32.56	0.055	0.139	0.040
Villus width (μm)	145.24 ± 12.24^b^	166.97 ± 4.71^a^	144.62 ± 5.66^b^	144.04 ± 5.05^b^	3.95	0.107	0.449	0.149
Crypt depth (μm)	299.36 ± 13.31^a^	308.21 ± 16.33^a^	242.85 ± 8.07^b^	270.18 ± 14.06^ab^	7.60	0.005	0.014	0.491
Intestinal wall thickness (μm)	303.58 ± 27.70	340.50 ± 27.80	287.04 ± 14.62	329.69 ± 23.66	12.04	0.398	0.818	0.906
VH/CD	3.34 ± 0.18^b^	4.10 ± 0.28^ab^	4.92 ± 0.40^a^	4.37 ± 0.38^a^	0.18	0.013	0.010	0.052

^a,b^Means in the same row with different superscripts differ significantly (*p* < 0.05). Values are mean, *n* = 10 hens per treatment.

As shown in Table 7, ileal intestinal wall thickness (linear, *p* < 0.05) increased with increasing MBP supplementation. Compared with the CON group, the TRE1 and TRE3 groups exhibited significantly increased ileal intestinal wall thickness (*p* < 0.05). The ileal villus width in the TRE1 group was significantly higher than that in the TRE3 group (*p* < 0.05), but did not differ significantly from that in the CON group (*p* > 0.05). No significant differences were observed among groups in ileal villus height, crypt depth, or VH/VD ratio (*p* > 0.05).

**Table 7:** Effect of MBP on ileal morphology of hens.

Items	CON	TRE1	TRE2	TRE3	SEM	*P*-value		
	0%MBP	2%MBP	3%MBP	4%MBP		ANOVA	Linear	Quadratic
Villus height (μm)	834.66 ± 24.15	861.03 ± 28.86	792.23 ± 33.27	806.19 ± 63.63	19.93	0.638	0.401	0.879
Villus width (μm)	118.08 ± 3.99^ab^	128.78 ± 4.93^a^	115.99 ± 3.26^ab^	109.73 ± 5.66^b^	2.45	0.042	0.071	0.071
Crypt depth (μm)	179.56 ± 9.44	174.12 ± 6.27	172.28 ± 9.85	176.59 ± 7.45	4.05	0.934	0.776	0.565
Intestinal wall thickness (μm)	280.44 ± 9.98^b^	326.82 ± 12.60^a^	315.33 ± 13.00^ab^	337.92 ± 17.72^a^	7.39	0.030	0.012	0.338
VH/CD	4.78 ± 0.31	5.02 ± 0.28	4.77 ± 0.38	4.61 ± 0.39	0.17	0.865	0.620	0.570

^a,b^Means in the same row with different superscripts differ significantly (*p* < 0.05). Values are mean, *n* = 10 hens per treatment.

### Effect of MBP on cecal microbiota diversity

**Figure 1:**
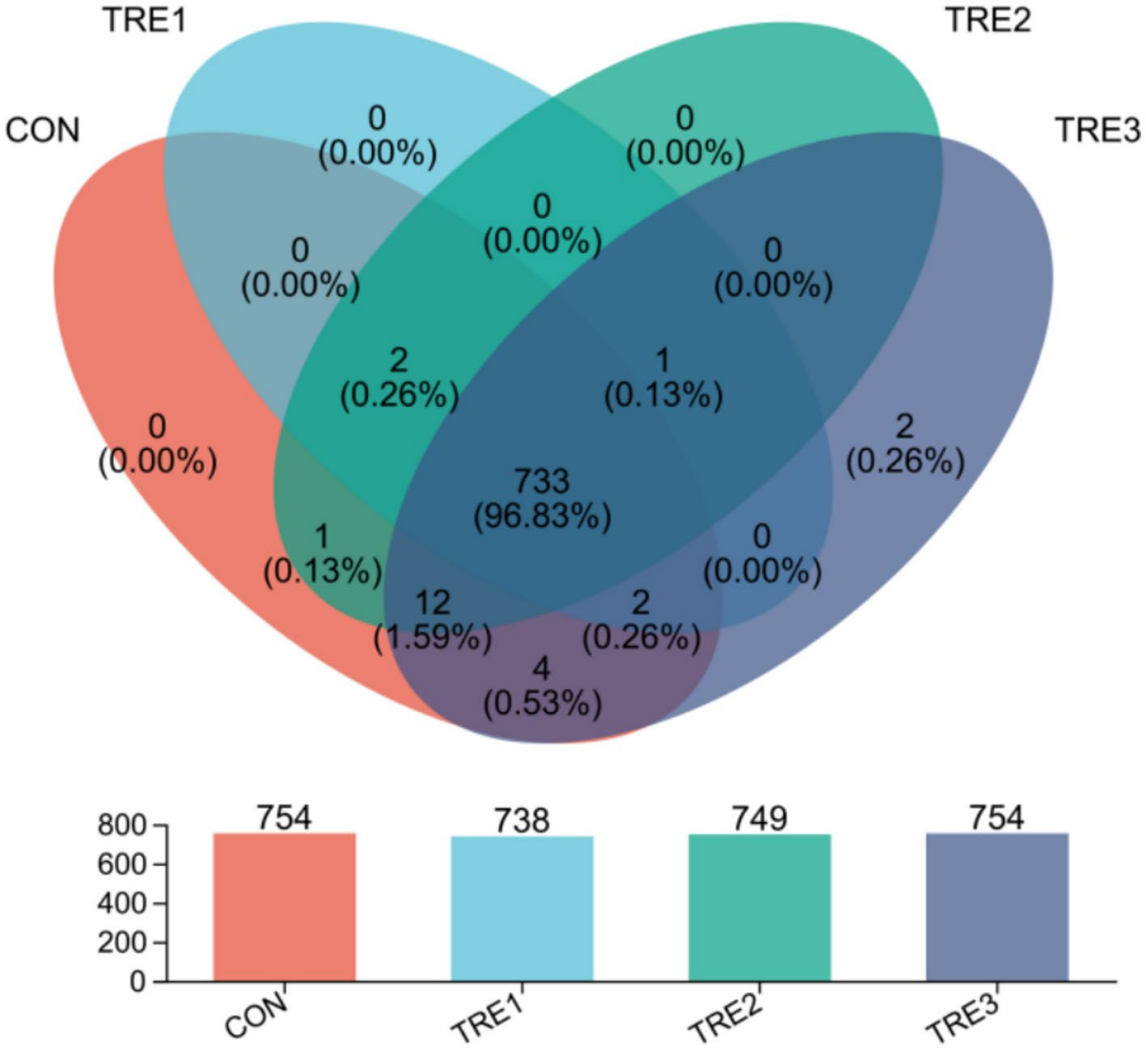
Venn diagram of cecal microbial OTUs in different experimental groups of hens.

To investigate the effects of dietary supplementation with MBP on the diversity and abundance of cecal digesta microbiota in hens, PCR amplification of the bacterial 16S rRNA V3–V4 region was performed for each sample, followed by high-throughput sequencing on the Illumina MiSeq platform. Statistical analysis of OTUs after rarefaction (Figure 1) showed that the control group contained 754 OTUs. The number of OTUs in the TRE1 and TRE2 groups was lower than that in the control group, with 738 and 749 OTUs, respectively. Compared with the control group, the number of unique OTUs in the TRE1 and TRE2 groups was one each. The number of OTUs in the TRE3 group was the same as that in the control group, with three unique OTUs. These results indicated that MBP had a minor effect on the regulation of OTU numbers in the cecal digesta microbiota of hens, and increasing inclusion levels may lead to greater differentiation in bacterial genera.

As shown in Table 8, dietary supplementation with MBP had a significant effect on the Sobs richness index (*p* < 0.05) and showed a tendency to alter the Ace (*p* = 0.059) and Chao (*p* = 0.071) indices of the cecal digesta microbiota in hens. The richness indices Sobs, Ace, and Chao in the TRE1 group were significantly lower than those in the CON and TRE2 groups (*p* < 0.05), and the Shannon diversity index in the TRE1 group was significantly lower than that in the TRE2 group (*p* < 0.05).

**Table 8:** Alpha diversity indices of cecal microbiota in different experimental groups of hens.

Items	CON	TRE1	TRE2	TRE3	SEM	*P*-value		
	0%MBP	2%MBP	3%MBP	4%MBP		ANOVA	Linear	Quadratic
Sobs	605.40 ± 6.64^a^	570.50 ± 11.45^b^	610.60 ± 6.47^a^	593.70 ± 10.88^ab^	5.05	0.018	0.904	0.332
Ace	650.19 ± 7.01^a^	624.40 ± 7.80^b^	653.72 ± 5.56^a^	644.58 ± 10.58^ab^	4.23	0.059	0.727	0.302
Chao	654.91 ± 8.20^a^	628.20 ± 8.91^b^	658.96 ± 7.45^a^	649.66 ± 9.63^ab^	4.54	0.071	0.698	0.317
Shannon	4.84 ± 0.06^ab^	4.71 ± 0.10^b^	4.93 ± 0.06^a^	4.80 ± 0.06^ab^	0.04	0.227	0.763	0.975
Simpson	0.021 ± 0.003	0.024 ± 0.004	0.017 ± 0.002	0.021 ± 0.002	0.001	0.353	0.538	0.993

^a,b^Means in the same row with different superscripts differ significantly (*p* < 0.05). Values are mean ± SE, *n* = 10 replicates per treatment.

**Figure 2:**
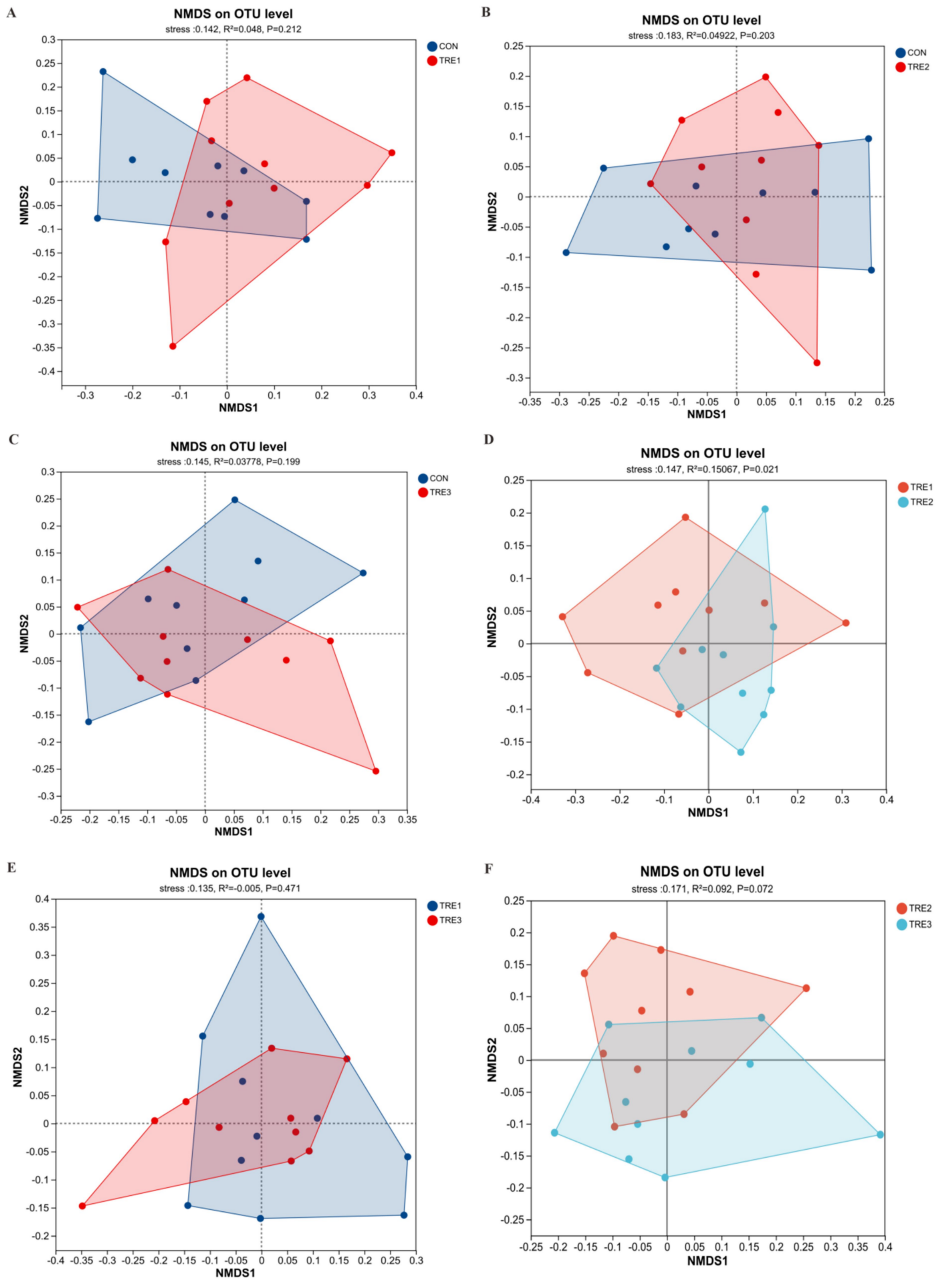
Beta diversity analysis of cecal microbiota in different experimental groups of hens. **A–F** shows PCoA plot based on Bray-Curtis distances between CON and TRE1 **(A)**, CON and TRE2 **(B)**, CON and TRE3 **(C)**, TRE1 and TRE2 **(D)**, TRE1 and TRE3 **(E)**, TRE2 and TRE3 **(F)**, with significance of community structure differences tested by ANOSIM.

To investigate the effect of dietary supplementation with MBP on the *β*-diversity of cecal digesta microbiota, non-metric multidimensional scaling (NMDS) was used to determine differences among groups. Principal coordinate analysis (PCoA) based on Br-Curtis distances calculated from OTU relative abundances (Figure 2) revealed a significant separation in microbial community structure between TRE1 and TRE2 (*p* < 0.05). A trend of separation in β-diversity was observed between the TRE2 and TRE3 (*p* < 0.10), which, given the lack of statistical significance, merits further validation with larger sample sizes. No significant separation was observed among the other groups.

### Effect of MBP on cecal microbiota composition

**Figure 3:**
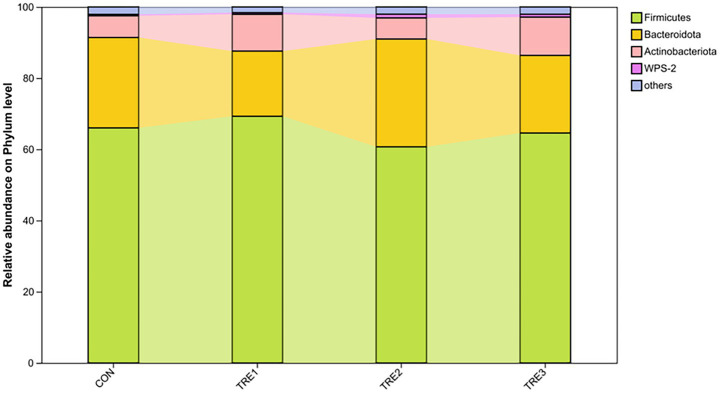
Microbial composition at the phylum level in cecal digesta of hens in different experimental groups.

**Figure 4:**
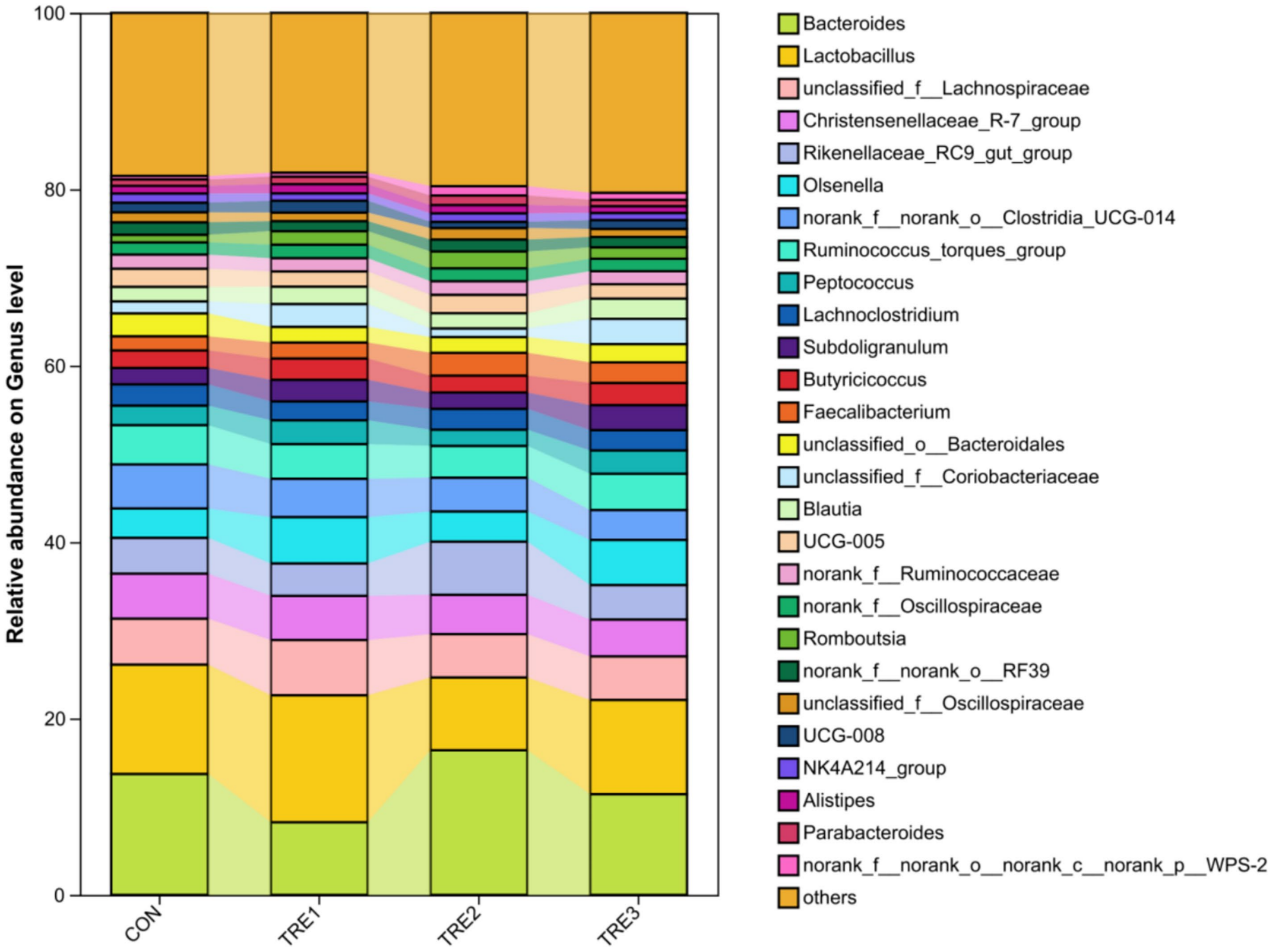
Microbial composition at the genus level in cecal digesta of hens in different experimental groups.

At the phylum level, the cecal microbiota composition of hens in different groups was shown in Figure 3. The dominant phyla in the cecum of hens were *Firmicutes, Bacteroidota, Actinobacteriota*, and *WPS-2*. At the genus level, the cecal digesta microbiota composition in different groups was presented in Figure 4. A total of 27 dominant genera were shared among groups, including *Bacteroides*, *Lactobacillus*, *unclassified_f_Lachnospiraceae*, and *Christensenellaceae_R-7_group*.

**Figure 5:**
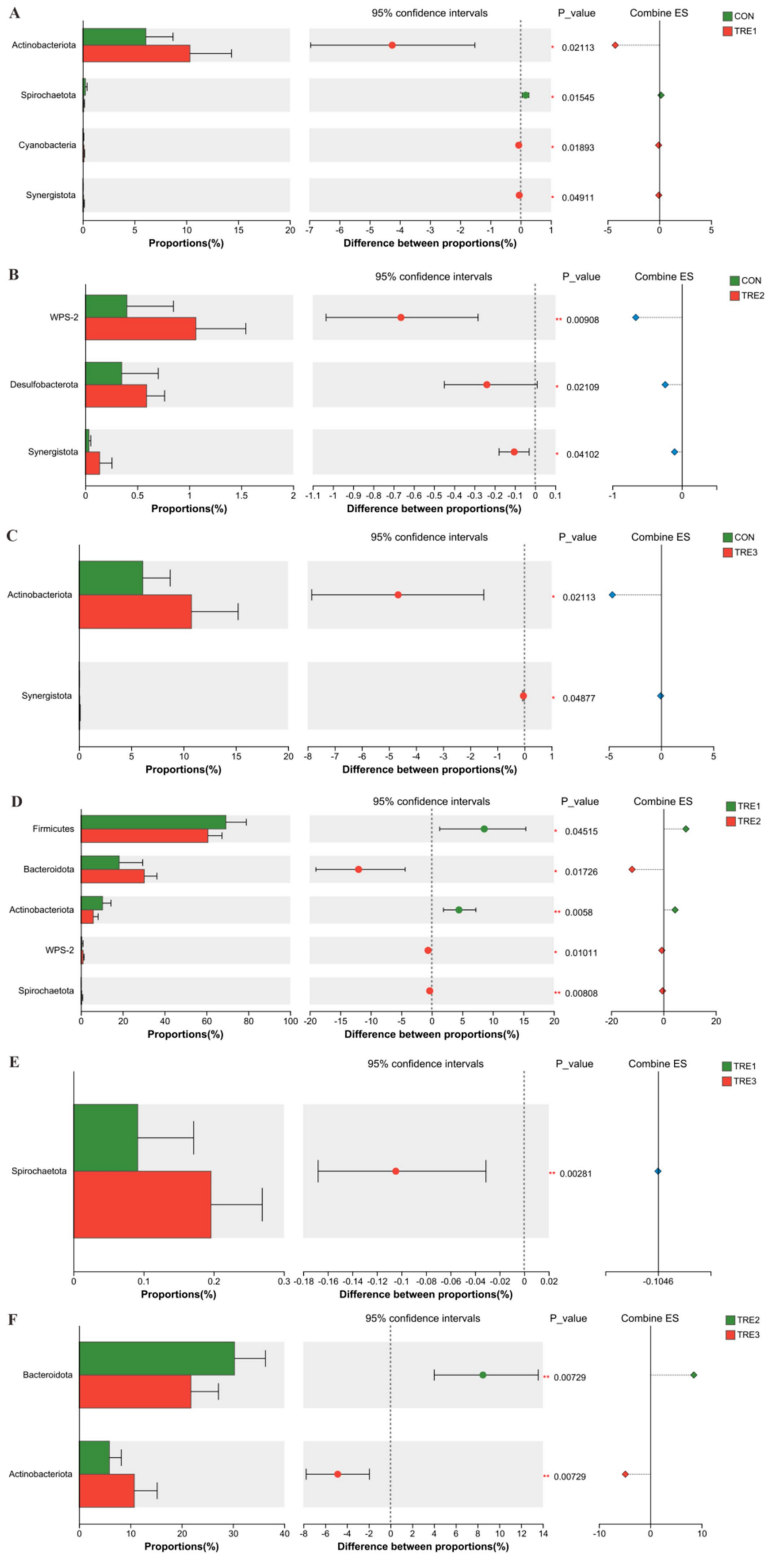
Differential microbial taxa at the phylum level in cecal digesta of hens in different experimental groups. Cecal digesta microbiota differed between CON and TRE1 **(A)**, CON and TRE2 **(B)**, CON and TRE3 **(C)**, TRE1 and TRE2 **(D)**, TRE1 and TRE3 **(E)**, TRE2 and TRE3 **(F)**.

Comparative analysis of differential microbiota at the phylum level among experimental groups, focusing on taxa with relative abundances exceeding 1%, was shown in Figure 5. Compared with the CON group, the relative abundance of *Actinobacteriota* in the TRE1 and TRE3 groups was significantly increased (*p* < 0.05), and the proportion of *WPS-2* in the TRE2 group was significantly increased (*p* < 0.05). Compared with the TRE1 group, the TRE2 group exhibited significantly lower proportions of *Firmicutes* and *Actinobacteriota* (*p* < 0.05), whereas the proportion of *Bacteroidota* was significantly increased (*p* < 0.05). Compared with the TRE2 group, the TRE3 group had a significantly lower proportion of *Bacteroidota* and a significantly higher proportion of *Actinobacteriota* (*p* < 0.05).

**Figure 6:**
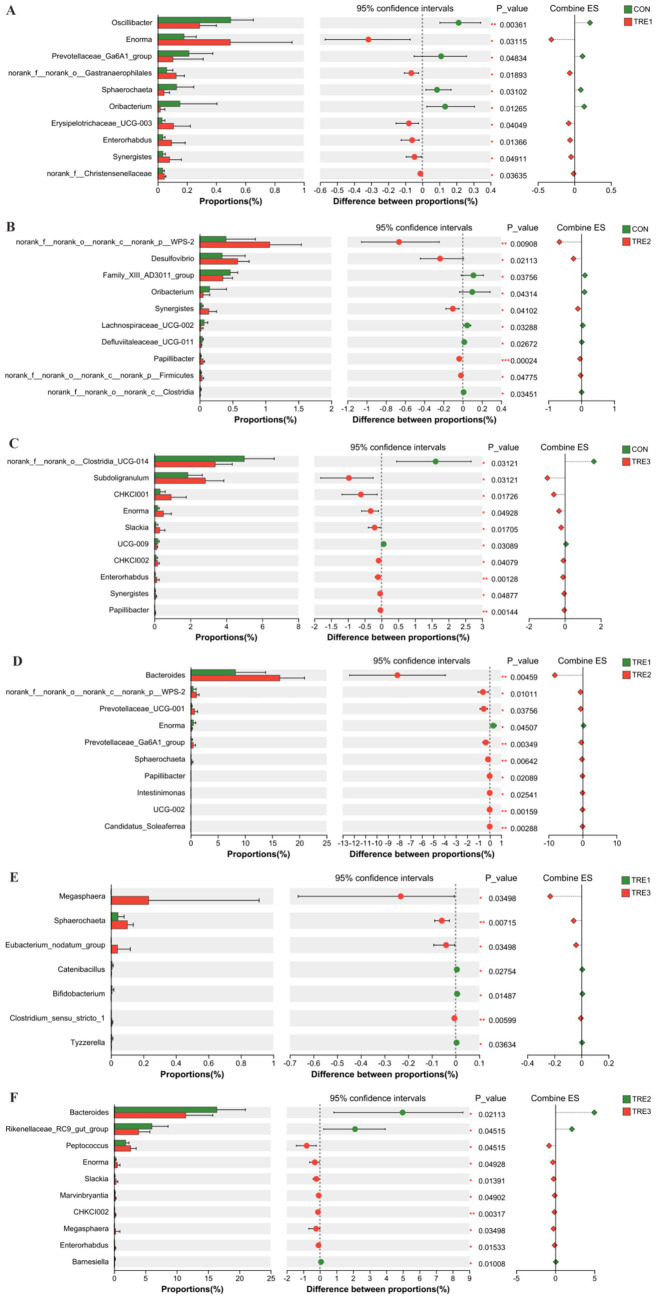
Differential microbial taxa at the genus level in cecal digesta of hens in different experimental groups. Cecal digesta microbiota differed between CON and TRE1 **(A)**, CON and TRE2 **(B)**, CON and TRE3 **(C)**, TRE1 and TRE2 **(D)**, TRE1 and TRE3 **(E)**, TRE2 and TRE3 **(F)**.

Comparative analysis of differential microbiota at the genus level among experimental groups, focusing on taxa with relative abundances exceeding 1%, was presented in Figure 6. Compared with the CON group, the relative abundance of *WPS-2* in the TRE2 group was significantly increased (*p* < 0.05), while the proportion of *Clostridia_UCG-014* in the TRE3 group was significantly decreased (*p* < 0.05). Compared with the TRE1 group, the TRE2 group showed a significant increase in the proportion of *Bacteroides* (*p* < 0.05). Compared with the TRE2 group, the TRE3 group exhibited significantly lower proportions of *Bacteroides* and *Rikenellaceae_RC9_gut_group* (*p* < 0.05).

When applying the more stringent LEfSe method (LDA > 2.0), no taxon at either the phylum or genus level reached the predefined threshold for differential abundance among the four groups. This suggests that the taxonomic shifts induced by MBP, while detectable by pairwise *t*-tests with FDR correction, did not exhibit sufficiently large effect sizes to be captured by the highly conservative LEfSe algorithm, likely due to the relatively moderate magnitude of compositional changes or the limited sample size per group (*n* = 10).

### Correlation analysis of gut microbiota with laying performance, intestinal morphology, and nutrient digestibility

**Figure 7:**
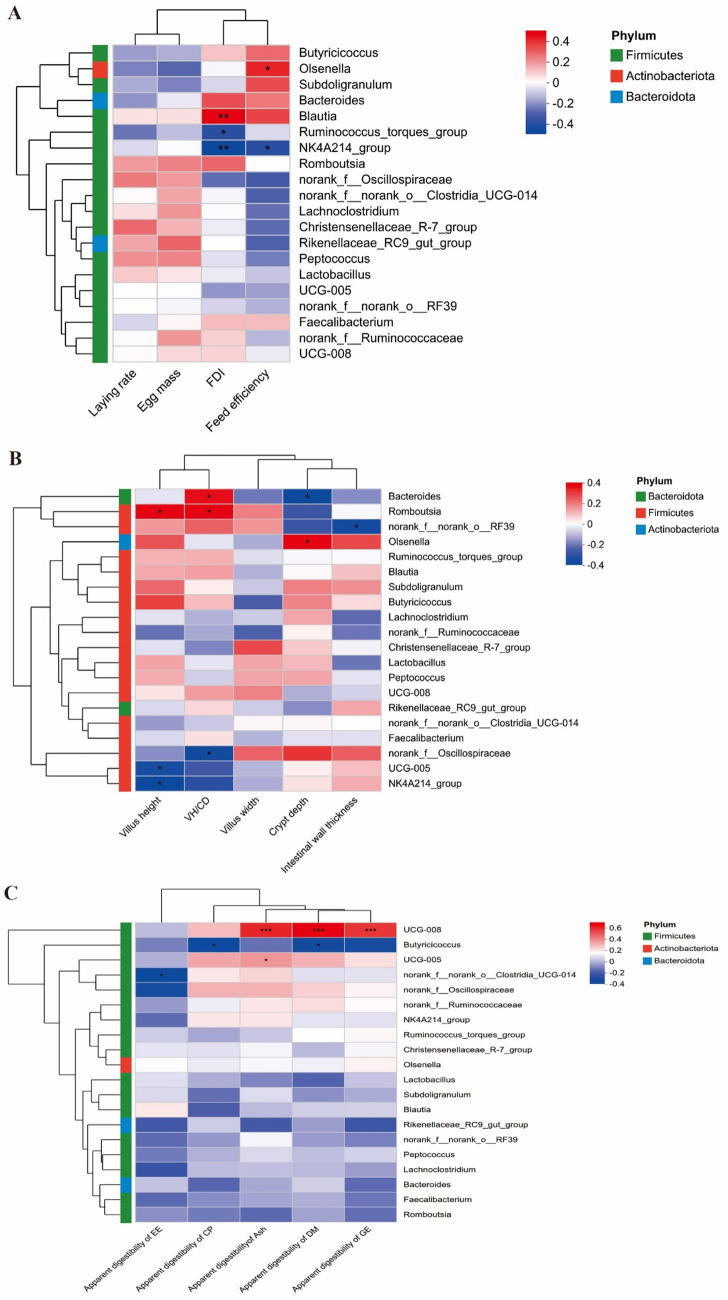
Heatmap showing correlations of cecal microbiota and laying performance **(A)**, jejunal morphology **(B)** and apparent nutrient digestibility **(C)**. The color in each block represents the Spearman correlation coefficient. *, ** and *** represent the *p <* 0.05, *p <* 0.01 and *p <* 0.001, respectively.

Correlations of gut microbiota and laying performance, jejunal morphology and apparent nutrient digestibility was performed in Figures 7A–C, respectively. The abundances of *Ruminococcus_torques_group* and *NK4A214_group* in the *Firmicutes* phylum were siginificantly negatively correlated with average daily feed intake (*p* < 0.05), whereas *Blautia* showed a significant positive correlation with FDI (*p* < 0.05). Meanwhile, the abundance of *NK4A214_group* in the *Firmicutes* phylum (*p* < 0.05) was significantly negatively correlated with feed efficiency. Conversely, the abundance of *Olsenella* in the *Actinobacteriota* phylum was significantly positively correlated with feed efficiency (*p* < 0.05). As shown in Figure 7B, the abundances of *Bacteroides* and *Romboutsia* were significantly positively correlated with villus height and VH/VD (*p* < 0.05), whereas *UCG-005* and *NK4A214_group* showed a significant negative correlation with villus height (*p* < 0.05), and *Oscillospiraceae* showed a significant negative correlation with VH/VD (*p* < 0.05). As shown in Figure 7C, the abundance of *UCG-008* was significantly positively correlated with apparent digestibility of DM, Ash and GE (*p* < 0.05), whereas *Butyricicoccus* showed a significant negative correlation with apparent digestibility of CP and DM (*p* < 0.05).

## Discussion

As a lignocellulosic feed ingredient rich in IDF, the appropriate inclusion level of MBP is critical for intestinal health and performance in laying hens. The results of this study demonstrated that the inclusion level of MBP in the diet exerted a significant dose-dependent regulatory effect on the laying performance. Compared with the control group, the 4% MBP supplementation group showed a significant decrease in laying rate during 15–28 d and over the entire experimental period (1–28 d), whereas no significant differences were observed in the 2 and 3% supplementation groups. Moreover, the 4% MBP group also exhibited a significant reduction in egg mass during both the later stage and the whole experimental period, further confirming the adverse impact of excessive MBP on overall egg production. This finding is consistent with the results of other studies on the application of IDF-rich ingredients in laying hens. It has been reported that supplementation with 6% wheat bran (primarily IDF) in aged laying hens significantly reduced average daily egg mass, whereas 3% inclusion had no adverse effects (Abdollahi et al., 2021). Similarly, another study found that increasing dietary crude fiber from 4.39 to 7% by adding rice bran and sunflower meal to laying hen diets significantly decreased egg weight and eggshell strength (Panaite et al., 2021). Collectively, these studies indicate that excessive inclusion of fiber ingredients may negatively affect laying performance and egg quality in laying hens, primarily through energy dilution and reduced available nutrient concentrations associated with high fiber levels (Morgan et al., 2022). Another study further confirmed that replacing 20% corn with 20% wheat bran in laying hen diets decreased feed efficiency, while additional supplementation with 2.5% soybean oil effectively alleviated this negative effect, suggesting that nutritional compensation is an important strategy to mitigate the adverse effects of high fiber (Novela et al., 2023). In summary, the negative effects of MBP in laying performance exhibited a clear dose-dependent pattern. It is recommended that the inclusion level of MBP should not exceed 3%. When inclusion exceeds this level, particular attention should be paid to its potential adverse effects on intestinal health and nutrient utilization. Meanwhile, this might also be associated with the reduced nutrient intake of laying hens resulting from the substitution of corn with MBP in the present study. The significant linear decrease in the analyzed AME values with increasing MBP indicated that energy dilution might contribute to the reduced performance at 4% MBP. In future research, dietary nutrient levels could be balanced based on the MBP nutrient database, allowing the functional regulatory effects of MBP to be further investigated. Furthermore, the bioactive components in MBP warrant further attention. It has been reported that bamboo shoot oligosaccharide extracts improved the reduction in yolk percentage and increased eggshell percentage in laying hens challenged with dextran sodium sulfate (Sittiya and Nii, 2024). Future research should explore the impact of micronization processing technology on bioactive components such as oligosaccharides in bamboo powder to provide a more comprehensively understanding of the mechanisms by which it modulates performance in laying hens.

Serum biochemical indices reflect, to a certain extent, the metabolic state of the organism, and the composition of dietary nutrients exerts a decisive impact on the nutritional metabolism of livestock and poultry. It has been shown that a high-fat, low-dietary fiber diet significantly upregulated the mRNA expression of glucose transporters and genes involved in the tricarboxylic acid cycle in the small intestine of broilers, thereby increasing serum glucose concentration while decreasing serum triglyceride content (Jiang et al., 2024). Furthermore, dietary fiber could effectively lower serum glucose levels by delaying gastric emptying rate (Muangchan et al., 2022). In the present study, although treatments did not significantly affect serum glucose levels, the serum glucose concentrations in the 3 and 4% MBP groups were numerically reduced by more than 10% compared with the CON group. Given the importance of glucose homeostasis for energy metabolism and laying performance in hens, this magnitude of reduction may hold potential practical value. Given that no significant differences in feed intake were observed among the groups in this experiment, it is speculated that this glucose-lowering effect is primarily associated with the reduced capacity of the intestine for glucose absorption and transport induced by the high-fiber diet. As a potential functional fiber feed ingredient, the metabolic regulatory effects of bamboo fiber have garnered increasing attention. It has been reported that replacing wheat bran with fermented bamboo fiber increased serum high-density lipoprotein cholesterol levels in growing-finishing pigs (Liu et al., 2022), suggesting a potential role in regulating fat metabolism. Similarly, supplementation with fermented bamboo shoot processing waste in the diet of weaned piglets significantly reduced serum triglyceride and urea nitrogen levels (Huang et al., 2022); perinatal supplementation with bamboo powder in sows significantly reduced serum total cholesterol and triglyceride concentrations (Dai et al., 2023); and dietary with wheat bran significantly reduced serum total cholesterol and triglyceride concentrations in broiler (Shang et al., 2020). However, in the present study, MBP had no significant effects on serum triglycerides, total cholesterol, or total protein levels in laying hens. In summary, under the conditions of this experiment, dietary supplementation with MBP induced a decrease in serum glucose concentration in laying hens but had limited regulatory effects on basal protein and lipid metabolism. This might be attributed to the physiological homeostatic regulatory capacity of laying hens to maintain laying performance.

The type and inclusion level of fiber ingredients are key factors affecting dietary nutrient digestibility. It has been reported that supplementation with 3% sugar beet pulp (primarily SDF) in laying hen diets significantly increased the ileal digestibility of dry matter, while supplementation with 3% wheat bran (primarily IDF) had no significant effect on the ileal digestibility of dry matter, organic matter, crude protein, or ash (Abdollahi et al., 2021). Another study showed that when formulating diets with crude fiber levels ranging from 2 to 8% by adding different levels of soybean hulls (primarily IDF), the digestibility of crude protein, crude fat, and crude fiber in laying hens increased with increasing fiber level, however, when the crude fiber content reached 11%, the digestibility of all nutrients decreased significantly (Zhang et al., 2023). Furthermore, broilers fed a diet formulated with purified cellulose (an IDF model ingredient) at 6% crude fiber exhibited significantly higher dry matter digestibility than those fed a diet formulated with soybean hulls (primarily IDF) at 8% crude fiber (Tejeda and Kim, 2020). Collectively, these studies suggest that, compared to SDF ingredients, high proportions of IDF ingredients are more likely to reduce dietary nutrient digestibility, and the extent of this effect varies among different IDF ingredients. In the present study, supplementation with 4% MBP significantly reduced the digestibility of dry matter, ash, crude protein, and gross energy in laying hen diets, whereas no significant differences were observed for any of these parameters at the 2% supplementation level compared with the control group. This indicates that the negative effect of MBP on nutrient digestion may be closely related to its inclusion level and that interference with nutrient utilization in laying hens is limited at lower inclusion levels. As a highly lignified source of IDF, improvements in its application potential in laying hen diets in the future could involve extracting and purifying bamboo-derived IDF through enzymatic hydrolysis or other methods based on micronization processing. Relevant studies have confirmed that extracted and purified bamboo-derived IDF can promote *in vitro* fermentation and increase the production of total short-chain fatty acids (Ge et al., 2022), providing a theoretical basis for its functional application.

The regulation of intestinal health is a central topic in dietary fiber nutrition research. Dietary fiber, particularly the IDF fraction, exerts a positive stimulatory effect on the development of intestinal morphological in poultry. It has been shown that the adding of lignocellulose or soybean hulls to low-fiber diets for broilers effectively increased ileal villus height and improve performance (Liebl et al., 2022). However, this promoting effect is dependent on fiber type and dosage. Another study has found that supplementation with 3% wheat bran to laying hen diets significantly increased jejunal villus height, VH/CD ratio, and villus surface area, whereas increasing the inclusion level to 6% or substituting with 3% or 6% sugar beet pulp did not yield this beneficial effects (Abdollahi et al., 2021). Interestingly, increasing the SDF content of fiber ingredients by modifying also promotes intestinal tissue development in poultry. Enzymatic modification of corn straw to increase SDF content significantly increased villus height and VH/CD ratio in the jejunum and ileum of laying hens (Wang et al., 2025). Collectively, the above studies confirm that the regulatory effects of fiber nutrition on intestinal morphology depends on its type, level, and physicochemical properties. In the present study, the 2 and 3% supplementation groups exhibited significantly increased jejunal villus height, which might partially offset the direct negative effects of high fiber levels on dietary nutrient digestibility, explaining why the production performance was maintained in the low-level MBP groups. Interestingly, this study also found that the 4% MBP group showed significantly increased intestinal wall thicknesses in the duodenum and ileum, which might be partly explain the observed reduction in nutrient digestibility. As a functional dietary fiber ingredient, bamboo fiber has been shown to possess bioactive properties. One study found that an SDF extracted from bamboo shoots significantly alleviated colonic pathological damage, inhibited the activation of inflammatory signaling pathways, and restored intestinal barrier function (Li et al., 2022). This suggests that bamboo fiber may possess bioactive regulatory functions beyond mere mechanical stimulatory effects on the intestine. Therefore, future research should focus on the deep processing of bamboo fiber to optimize its physicochemical properties and biological activities, aiming to better realize its potential to promote intestinal health while ensuring nutrient utilization.

The gut microbiota is considered a crucial link connecting dietary components with host health and nutrient metabolism. This study focused on analyzing the effects of varying levels of MBP on the cecal microbial diversity and community structure in laying hens. Current findings regarding the regulation of cecal microbial diversity by dietary fiber in laying hens are inconsistent. A study has shown that supplementation with wheat bran or purified cellulose (an IDF model ingredient) increased cecal microbial *α*-diversity in laying hens, primarily reflected by an increase in the Shannon index (de Sousa et al., 2025). In contrast, another study has found that dietary with 2, 3, 4, and 5% mulberry branch fiber significantly reduced the α-diversity (Chao1 and Shannon indices) of cecal digesta (Hu et al., 2024). Similarly, our previous research demonstrated that supplementation with 1% MBP in broiler diets significantly reduced the Faith_pd index and tended to reduce the Chao1 index of cecal digesta (Dai et al., 2022). The present study further confirmed the significant effects of MBP on the cecal microbial α-diversity in laying hens. Specifically, the richness indices (Sobs, Ace, Chao) of cecal digesta microbiota in the 2% MBP group were significantly lower than those in the control group, whereas no significant differences were observed between the 3% or 4% MBP groups and the control group. This suggests that dietary supplementation with MBP does not simply reduce microbial richness but may drive a structural reshaping of the microbial community in a dose-dependent manner. In the present study, we processed 16S rRNA data using an OTU-based pipeline (UPARSE, 97% identity). Although this approach remains widely used and suitable for our comparative analyses, ASV-based methods (e.g., DADA2, Deblur) offer higher taxonomic resolution and reproducibility. Accordingly, the taxonomic assignments reported here should be interpreted at the OTU level, and future studies employing ASV are warranted to refine these findings.

Further analysis in this study revealed that at the phylum level, the dominant cecal bacteria across all groups consisted of *Firmicutes*, *Bacteroidota*, *Actinobacteriota*, and *WPS-2*, which is largely consistent with previous reports on the cecal microbiota structure of laying hens (Xu et al., 2023). Among these, *Actinobacteriota*, a core beneficial bacterial group in the cecum of laying hens, plays a critical role in fiber degradation and the maintenance of intestinal homeostasis (Lewin et al., 2016). It has been shown that supplementation with a fructan-rich root extract significantly increased the proportion of cecal *Actinobacteriota* and reduced egg weight loss during storage, thereby improving egg quality (Wu et al., 2024). Our results are similar, with the relative abundance of cecal Actinobacteriota being significantly higher in the 2 and 4% MBP groups compared to the control group. Similar results were observed in the present study, where the relative abundance of *Actinobacteriota* in the cecal digesta of the 2 and 4% MBP groups was significantly higher than that in the control group. This increase in abundance may be attributed to the adaptive fermentation requirements associated with the complex fiber components in MBP. The proliferation of *Actinobacteriota* likely enhances the fermentative capacity of the hindgut, leading to increased production of short-chain fatty acids, which provide energy to the host and improve the intestinal environment (Hu et al., 2021). This may represent a key mechanism underlying the biological value of bamboo fiber as a functional feed ingredient. At the genus level, the present study found that the relative abundance of *WPS-2* in the cecal digesta was significantly lower in the 3% MBP group compared with the control group, whereas the relative abundance of *Clostridia_UCG-014* was significantly lower in the 4% MBP group. *WPS-2* represents a recently identified group of bacteria that includes various metabolically active species, and its presence as a dominant cecal genus has also been observed in late-laying hens supplemented with kaempferol and vitamin E (Zhang et al., 2024). A reduction in the abundance of *Clostridia_UCG-014* has been considered a potential marker of gut dysbiosis in some studies; for instance, mice with dextran sulfate sodium-induced colitis exhibited a significant decrease in intestinal *Clostridia_UCG-014* abundance, whereas supplementation with curcumin restored its abundance and improved colonic morphology (Deng et al., 2024). In summary, appropriate supplementation with MBP in laying hen diets may enhance hindgut fermentation capacity by promoting metabolic functional bacteria, thereby partially counteracting the negative effects of fiber ingredients on nutrient digestibility and sustaining laying performance. In contrast, excessive supplementation with MBP might alter community composition, leading to increased intestinal wall thickness and reduced nutrient digestibility.

It was worth noting that while pairwise *t*-tests with FDR correction identified several phylum and genus level differences among groups, the more stringent LEfSe method (LDA > 2) did not confirm these as differentially abundant. This discrepancy might be attributed to the inherently higher stringency of LEfSe, which required both statistical significance and a large effect size. Given the relatively moderate shifts in cecal microbiota composition and the limited sample size (*n* = 10) in the present study, it was not unexpected that only taxa with very strong effect sizes would pass the LEfSe threshold. Importantly, the absence of LEfSe-confirmed differences did not invalidate the FDR-corrected *t*-test findings, rather, it highlighted that the observed microbial changes were subtle and might require larger sample sizes or more targeted approaches to detect robust effect sizes. We therefore interpreted the FDR-corrected *t*-test results as exploratory indicators of MBP-associated microbial shifts, warranting further validation in future studies with increased statistical power.

The modulatory effects of lignocellulosic fibers on gut microbiota have been documented in laying hens. It has been shown that dietary with 5% mulberry branch fiber (a lignocellulosic ingredient primarily composed of IDF) significantly increased the abundance of microbial taxa involved in fatty acid degradation and SCFAs biosynthesis in the intestine of laying hens via the enterohepatic axis, thereby reducing liver fat content and improving the fatty acid composition of egg yolk (Hu et al., 2024). In contrast, another study found that as the dietary inclusion level of ryegrass increased, the digesta passage rate in laying hens increased linearly, whereas the apparent metabolizable energy and apparent digestibility of dry matter and NDF decreased linearly. And supplementation with exogenous enzymes moderately improved the digestibility of crude protein and NDF but had no significant effect on apparent metabolizable energy (Suyama et al., 2025). These studies highlight that the response to IDF is highly dependent on fiber source, inclusion level, and processing method – a concept that aligns with our dose-dependent findings with MBP, including laying performance, apparent nutrient digestibility and intestinal morphology.

Correlation analyses in this study provided further insights into the functional implications of MBP-induced microbial changes. The *NK4A214_group* belongs to the family *Ruminococcaceae* and has been associated with fiber degradation and ruminal fermentation parameters (Pang et al., 2022). It has been found that the relative abundance of *Ruminococcaceae_NK4A214_group* was negatively correlated with milk yield (Dong et al., 2023). In the present study, the negative correlations between *NK4A214_group* abundance and feed intake, feed efficiency and villus height, suggest that excessive proliferation of this genus under high-fiber conditions may compromise rather than enhance nutrient utilization, possibly through excessive fermentation activity that competes for energy substrates or through indirect effects on host appetite regulation. *Blautia* has been identified as a promising probiotic for maintaining colonic mucus function under low-fiber consumption through secretion of short-chain fatty acids (Holmberg et al., 2024). The positive correlation between *Blautia* abundance and daily feed intake in this study is therefore consistent with its known beneficial roles in gut health and nutrient absorption. The genus *UCG-008* has been identified as a potential marker for intestinal health and metabolic regulation (He et al., 2025). Another study has found that oat bran supplementation increased the abundance of *Butyricicoccus* while simultaneously reducing the digestibility of gross energy, dry matter, and crude protein (He et al., 2018). In the present study, the abundance of *UCG-008* was significantly positively correlated with apparent digestibility of DM, Ash and GE, whereas *Butyricicoccus* showed a significant negative correlation with apparent digestibility of CP and DM. The integration of correlation analyses in this study provides a more comprehensive understanding of the host-microbiota interactions underlying the dose-dependent effects of MBP on laying hen performance.

## Conclusion

The regulatory effects of MBP on laying performance, intestinal health, and nutrient digestibility in laying hens exhibited a clear dose-dependent pattern. Appropriate supplementation (2–3% MBP) as a partial substitute for corn maintained laying performance by improving intestinal morphology and optimizing cecal microbial community structure, thereby realizing the potential benefits of “substituting bamboo for grain.” However, high-level supplementation (4%) induced intestinal wall thickening, significantly reduced nutrient digestibility, and altered community composition, ultimately leading to decreased laying performance. Future studies should not only formulate diets balanced for energy based on a comprehensive MBP nutrient database to disentangle functional effects from energy dilution, but also employ extended feeding periods (e.g., 12 weeks) to evaluate the sustainability of the beneficial effects of MBP and confirm the absence of adverse effects over a full laying cycle. Providing one of the first evaluations of bamboo fiber in layers, this study elucidates preliminary mechanisms and establishes a recommended maximum dietary inclusion level of 3% MBP.

## Data Availability

The original contributions presented in the study are publicly available. This data can be found at: https://www.ncbi.nlm.nih.gov, accession number PRJNA1430196.

## Footnotes

1
www.majorbio.com

2 https://github.com/OpenGene/fastp, version 0.19.6.

3 http://www.cbcb.umd.edu/software/flash, version 1.2.11.

4 http://drive5.com/uparse/, version 7.1.

5
https://cloud.majorbio.com

6
http://www.mothur.org/wiki/Calculators
